# Practical Principles of Palliative Care in Veterinary Oncology: Alleviating the Suffering of the Animal, Owner, and Veterinarian

**DOI:** 10.1155/2024/5565837

**Published:** 2024-07-13

**Authors:** Beatriz F. Paz, Marília G. P. A. Ferreira, Ketlyn R. Martins, Lucas Uccella, Andrigo Barboza de Nardi

**Affiliations:** ^1^ Programa de Pós-Graduação em Ciências Veterinárias Faculdade de Ciências Veterinárias Universidade Estadual Paulista, 14.883-000, Jaboticabal, São Paulo, Brazil; ^2^ Hospital Veterinário Universitário da Universidade Federal do Vale do São Francisco, 56.340-000, Petrolina, Pernambuco, Brazil; ^3^ Faculdade de Ciências Veterinárias Universidade Estadual Paulista, 14.883-000, Jaboticabal, São Paulo, Brazil

## Abstract

**Background:**

Palliative care for pets is a developing area that encompasses animals with cancer and other chronic and degenerative diseases.

**Objectives:**

To elucidate the guiding principles of palliative care in veterinary oncology.

**Methods:**

This article was structured from an extensive literature review and addresses the growing need for improved palliative care in veterinary medicine. Methods of quality of life assessment in animals with cancer, owner education, and the importance of an interdisciplinary team approach are discussed.

**Results:**

Palliative care aims to improve the animal's quality of life, alleviating its physical, emotional, and social suffering. Through attentive communication, palliative care also seeks to alleviate owners suffering from the moment of diagnosis to mourning the patient's death.

**Conclusions:**

The importance of offering palliative care alongside anticancer techniques and treatments should not be underestimated and should ideally use a multidisciplinary team approach.

## 1. Introduction

Palliative care for animals aims to optimize patient comfort and minimize suffering, while also supporting the owner. It promotes the treatment of pain and other clinical signs to improve quality of life, regardless of disease development. This form of care considers the animals' social, emotional, and physical needs alongside the emotional, social, and spiritual needs of owners, preparing them for the animal's death and the guardian's grief [1, 2].

In human medicine, palliative care is already an integral part of end-of-life care, while in veterinary medicine, the concept is still developing. Veterinarians and pet owners have recently recognized this need and, over the past ten years, many veterinarians have shifted their focus to dedicate themselves to end-of-life care, some acting exclusively in these services [3, 4].

In veterinary medicine, cancer represents the leading cause of mortality and morbidity in elderly dogs and cats [5]. Marked advances in the diagnosis and treatment of cancer patients have been made over recent years, and a focus on improving the quality of life of animals with cancer is equally important [6].

Various treatment options are available to enable veterinarians to improve quality of life and survival times of animals with cancer, and this should include maintenance of a good relationship between animals and their owners [7]. The treatment of animals with cancer consists of several antitumor therapies, and palliative care is often only considered in the transition period between the interruption of cancer therapies and the end of life [3]. However, in human palliative care, the importance of providing such care early on after the diagnosis of cancer is noteworthy [8].

In order to provide optimal palliative care, it is acknowledged that an interdisciplinary approach is essential [9]. Currently, many veterinarians have dedicated themselves to the practice of palliative and end-of-life care together with a network of auxiliary care providers that range from complementary therapy specialties, such as physiotherapy and acupuncture, to working together with social workers [10]. In this review, we seek to elucidate the guiding principles and practice of palliative care in veterinary oncology.

## 2. Materials and Methods

The article was prepared based on an extensive literature review conducted through research in databases such as PubMed, Bireme, SciELO, and Google Scholar. Its structure addresses the growing need to improve palliative care in veterinary medicine. The principles of palliative care are discussed within the context of the triad consisting of the animal, owner, and professional team. This includes methods for assessing the quality of life in animals with cancer, owner education, and the importance of an interdisciplinary team approach. In addition, the article provides a structured descriptive framework on the treatment of clinical cases available in the literature and treatment protocols. Finally, it addresses the paradigms and challenges of palliative care practice in veterinary medicine in a complementary manner.

## 3. Need to Introduce Palliative Care in Veterinary Medicine

The historical development of palliative care originated from the service of inns, places that offered rest and shelter to pilgrims who traveled towards sanctuaries, in which they sought to find a cure for their illnesses. From the 11th century onwards, some of these facilities began to provide specific care for incurable patients. However, it was mainly from the work of Dame Cicely Saunders in the second half of the 20th century that modern palliative care, as it is known today, unfolded [11]. Based on the idea of “total pain,” Saunders praised the importance of effective control, in its physical, emotional, social, and spiritual dimensions, and those who were faced with the physical, rigorous, and necessary treatment of patients [12].

Based on compassion and care for patients and families, there is a great need for palliative care in the current era of medicine [13]. In a survey carried out in 2017, 151 countries offered palliative care in an isolated or specialized manner in the area of human health through palliative care programs [14]. Even in the face of medical advances, patients with life-limiting diseases still suffer considerably [13]. In a systematic review, evidence from 38 studies indicated that an average of 33 to 38% of individuals near the end of life receive nonbeneficial or potentially harmful treatments [15].

Currently, palliative care is considered a growing medical specialty that provides specialized relief of symptoms and pain, as well as emotional support for patients and their families [16]. The World Health Organization (WHO) recognizes the provision of palliative care as an ethical responsibility of healthcare systems, and it is the moral duty of health professionals to alleviate pain and suffering, be they physical, psychosocial, or spiritual [17]. In this way, palliative care promotes a paradigm shift from the biomedical to the biopsychospiritual model [13]. According to Snaman et al., it has been noted that the integration of palliative care in the routine care of children, adolescents, and young adults with cancer fosters better outcomes for patients and their families [18].

However, the establishment of the practice and design of palliative care in veterinary medicine began relatively recently, in the late 1980s [4, 11, 16]. Veterinary palliative care (VPC) programs were formalized in the United States by the American Veterinary Medical Association (AVMA) after the publication of the Veterinary Hospice Care Guidelines in 2001, which were later revised by the institution in 2007 and 2016 [2, 11].

The first university program developed for palliative care in companion animals dates back to 2003. The College of Veterinary Medicine and Biomedical Sciences at Colorado State University (CSU) developed Pet Hospice, a support program for companion animals, caregivers, and veterinarians dedicated to educating students and professionals about end-of-life care [19].

In 2011, the College of Veterinary Medicine at Michigan State University (MSU) founded Veterinary Hospice Care, intended for the home care of companion animals. The hospice operated until 2014 with the objective of providing excellent service to patients in the final stage of life, as well as emotional support to families, approaching them as a care unit. During the first three years of activity, the service registered a total of 142 patients, which increased in number each year [20].

In 2019, Moses published a review regarding the Pain and Palliative Care Service of the Massachusetts Society for the Prevention of Cruelty to Animals (MSPCA)—Angell Animal Medical Center, whose work embraces the care of several species of companion animals, including birds, reptiles, and mammals [21]. In institutions where VPC was applied, a high number of cancer patients were observed. By the year 2019, 66% of patients treated by the CSU Pet Hospice Program were animals diagnosed with cancer [19]. In MSU's Veterinary Hospice Care, a similar number were observed, in which 79% of the enrolled patients had diagnoses or clinical signs compatible with neoplasms [20].

In addition to the application of palliative care in oncology, this area has grown in other branches of veterinary medicine, such as patients with acute and severe neurological diseases [22] and those requiring nutritional support, for whom adequate recommendations for assisted nutrition and hydration are critical in the final phase of life [23].

The presence of comorbidities is common in elderly animals with cancer. Osteoarticular and endocrine diseases, as well as neoplasms, may coexist. There are cases in which animals receive palliative chemotherapy alongside treatment guided by palliative care, as seen in the treatment of splenic lymphoma in a 12-year-old dog. This therapy involved adjusting chemotherapy doses to prevent side effects. In addition to adjusting antineoplastic treatment, nutritional support, treatment of paraneoplastic cachexia, pain management, and kidney disease treatment were important for improving the quality of life during patient care. End-of-life care planning ensured that owners received full veterinary support, resulting in a survival time of over one year [24].

Recently, the study and provision of palliative care in human oncology, considering a new and emerging field in science and clinical practice, has undergone significant development. Researchers have evaluated different models of palliative care in various populations while expanding palliative care programs. Several questions regarding the models of initial care delivery, the best way to integrate palliative care in different patient populations and how to implement high-quality programs with good cost-benefit ratios, continue to strongly motivate further studies [25]. The design of a VPC service requires structured planning, including the development of specific knowledge and skills, in addition to defining the service configuration and practice values. Many models and advanced training for this purpose have arisen from experiences in human palliative care services [21].

Although renowned veterinary institutions have already published articles on end-of-life care [2], there is still no title of specialist in palliative care. Faced with the increase in life expectancy and better access to information and resources, an increasing number of caregiver clients has been looking for treatments that improve quality of life, promoting the rise in interest in palliative care for companion animals, making it one of the most growing specialties demanded from animal owners in upcoming years [4, 6, 10, 11, 16].

Thus, veterinarians should be prepared to deliver humanistic care aimed at maintaining good relationships between professionals and their clients. In a study conducted in Switzerland in 2013, in which more than 2,000 animal owners who sustained the loss of a pet were asked whether they received grief support, 34% of the owners spoke with the veterinarian, 15% with the veterinary technician, while 2% had talked with no one [26]. However, in a survey carried out in 2013, among 119 Brazilian universities, none of the veterinary medicine curricula included any teaching on the provision of care for animal owners, and when dealing with matters such as animal welfare, the main focus was on humane euthanasia. Only one curriculum included psychology, which reinforces the gap in veterinary medical training regarding client communication, particularly concerning issues such as death and family well-being [27]. In contrast, 4–7% of veterinary schools in the USA address matters related to palliative care and end-of-life decisions, while in the United Kingdom, a survey involving 6 veterinary schools found that all addressed communication with owners regarding the death of animals in their curriculum, but only one (16.6%) addressed end-of-life decisions [28, 29].

Over the years, a correlation has been observed between the year of graduation and the level of preparation when approaching end-of-life issues, with recent graduates showing better preparation on the topic [30]. After accessing a structured course for training in palliative care, fourth-year US veterinary students said they felt more prepared for end-of-life conversations and euthanasia practice. In addition, they obtained positive results regarding professional well-being and showed a greater ability to deal with emotional issues arising from professional practice [31].

Even in the face of the delay concerning palliative care training, it is of paramount importance that veterinarians develop good communication skills regarding end-of-life issues and grief, since they are essential for the practice of palliative care [16].

Veterinary patients can receive treatment in emergency services, even when presenting with neoplastic diseases at advanced or metastatic stages, without the necessity of invasive tests for a definitive diagnosis. These patients require a thorough evaluation to identify possible underlying untreated conditions, such as osteoarticular pain. Palliative care planning is established through constant monitoring and adjustments of medication or therapies to provide better symptom control. In addition, adjunctive therapies such as anti-inflammatory drugs, anticonvulsants with analgesic properties, antioxidants, fish oil, and gastric protectors may be included in the treatment regimen. Other options, such as anxiolytics, cannabinoid therapy, antiemetics, and probiotics, among others, can also be considered [32].

Despite the growing number of articles addressing palliative care and end-of-life care protocols [2], research in this area appears to be incipient, with no published case studies found. Some authors have compared antineoplastic treatments with palliative treatments [33]. However, these comparisons often refer to an approach guided by principles of clinical oncology, without delving into the guiding principles of palliative care or considering individual therapeutic decisions. Similarly, the veterinary medicine curriculum does not provide sufficient training for professionals to offer palliative care, leading to dissatisfaction in more than 70% of respondents in a study [34]. The certification course promoted by the International Association of Animal Hospice and Palliative Care (IAAHPC) was cited by 42% of respondents as a source related to their professional development in care, along with the literature published in veterinary journals (40%) [34]. However, these sources mainly involve experiential reports [32, 35].

## 4. Guiding Principles for Palliative Care

**Figure 1:**
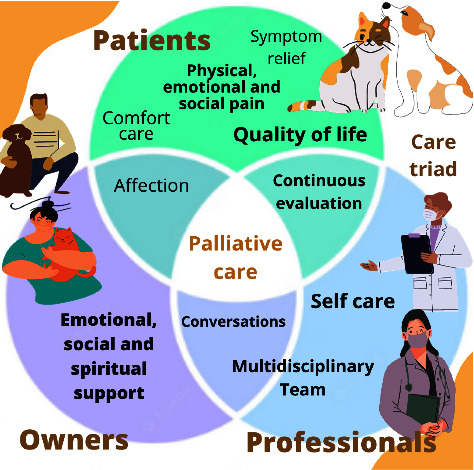
Principles of veterinary palliative care: the integration of specific care for the patient, professional, and owner.

The American Animal Hospital Association (AAHA) and the International Association for Animal Hospice and Palliative Care (IAAHPC) End-of-Life Care Guidelines [2] conceptualized hospice for animals as a need—care program that addresses the physical, emotional, and social needs of animals in advanced stages of progressive and life-limiting disease or disability from the time of diagnosis through to their death, including hospice-assisted natural death or death by euthanasia. It also addresses the human caregiver's social and emotional needs, preparing them for the death of the animal and the grief experience. Palliative care, in turn, is defined as the treatment of patients with curable or chronic clinical conditions with the aim of improving the quality of life of the animal and its caregiver [2]. The integration between the aspects of care aimed at the professional, patient, and owner is succinctly described in Figure 1.

In a review on the public perception of advanced care, palliative care, and human hospice, the authors noted that more than 9,000 survey participants had some difficulty understanding the concepts of palliative care and hospice [36]. Hospice applies to a more restricted group, being selected for patients in the final stage of life to ensure a “good death” and fits into a general discipline of palliative care [37].

Palliative care is aimed at the comfort and quality of life of the patient to the detriment of the quantity of life [16]. Several factors may justify the recommendation for palliative care and hospice, such as the decision not to perform treatments aimed at curing the disease, the diagnosis of a terminal illness, when curative treatment fails, the need for intensive care for an extended period, progressive disease or trauma with associated complications, or when there are symptoms of chronic diseases that interfere with the animal's functionality. In this context, it is recommended that palliative care or a hospice plan be started to promote a positive effect on the patient's end-of-life care [38]. Life-prolonging interventions such as cardiopulmonary resuscitation are declined if they no longer contribute to the patient's quality of life [2].

Some scientific papers focus on palliative care using the Pawspice philosophy approach, in which patients are treated in a more caring, gentle, and kind manner. Throughout this approach, the use of a quality-of-life assessment scale is recommended [39]. If the animal presents gradually worse in the scale scores, the veterinarian is encouraged to have a frank conversation with the owner regarding the expectations of the patient's condition so that euthanasia and palliative care-assisted natural death can be discussed and those responsible can plan and reflect on the best conduct that will benefit the caregiver and their animal [2, 39]. In addition, based on the Pawspice philosophy, the caregiver can decide to instruct not to resuscitate the patient in an emergency situation. This principle helps the family face the problem and avoid excessive and useless treatments for the animal [39].

Exploring and understanding the values surrounding palliative and end-of-life care has become imperative for the development of this service, helping mitigate the moral suffering of the caregiving team [21]. Veterinarians are often faced with the decision of how to treat their patients when aggressive treatment has been refused or is not in the animal's best interest. In this context, palliative care provides an important tool for treating animals during the period between diagnosis and the end of life [40].

Therefore, when treating a patient with cancer, the main concern on the part of the veterinarian should be the maintenance of the patient's quality of life. It is important for the client to be aware of available treatment options, likely outcomes, and the monitoring needed for therapy [6]. Palliative care complements antineoplastic treatment and can improve the therapeutic effect, optimizing the patient's general condition, as it acts in the management of symptoms and quality of life, thus improving the owner's adherence to treatment [41].

Palliative care can be subdivided into two categories, namely, early palliative care, which usually occurs in the outpatient or hospitalized patient setting, and late palliative care, which takes place in palliative care units, hospices, or the residence of patients already in the final stage of life [42].

In general, palliative care services can be conducted through home consultations, where nurses or assistants visit patients to administer medications, or through hospital services. The cost and time of care vary depending on the chosen modality, including expenses related to veterinary facilities, travel time, and team transport [32, 43]. Service fees should be determined based on the cost of materials and staff, taking into account regional variations [43]. In addition to the reported symptom improvement in clinical trials involving the inclusion of palliative care in the treatment of people with cancer, a significant reduction in the total cost of care and a decrease in the number of hospitalizations and intensive treatments for patients receiving palliative care have been observed [8, 44, 45].

Efficient communication in palliative care assists in the development of comprehensive and humanized care through active and empathetic listening, eye contact, and behavior, involving verbal and nonverbal language with the animals' owners [46]. In this way, the oncologist's role in providing emotional support to clients has expanded [3]. Recognizing the burden that clinical signs can cause to a caregiver of an animal with a chronic or terminal illness can facilitate communication between the veterinarian and the owner, as well as assist in planning and treatment [47].

End-of-life conversations for human patients are typically guided by the principles of a good death, being allowed to die, and defining when life-prolonging treatment is no longer appropriate. The patients' rights to suspend or withdraw support treatment for the maintenance of life are discussed, leading to ethical conflicts between the wishes of the patient, the family, and the caregiving team. In veterinary medicine, however, the conflict paradigm changes due to the wide acceptance of the practice of euthanasia. This is indicated as an option for patients in the final stage of life, also ensuring that other measures are taken to relieve discomfort, as focusing only on euthanasia can neglect the deep emotional legacy of grief [2, 48, 49]. When faced with a situation of euthanasia and acceptance of the procedure by the owner, veterinarians should consider arranging a prior consultation to ensure that any owner doubts concerning what will happen during and after the euthanasia are resolved preemptively [50].

In the veterinarian's relationship with clients whose animals have life-limiting diseases, providing open and honest information is crucial. In a qualitative study with owners of dogs with cancer, the results indicated the need for the professional to verify the caregiver's understanding and experiences, as well as their preferences for receiving news. The information provided gives the client the necessary confidence to get involved in the treatment, make informed decisions, and be prepared for the illness process, in addition to stimulating the perception of control, fostering hope, and improving the psychosocial well-being of the caregiver [51].

As such, goal-setting conversations on care are essential for palliative care since they provide a formal framework, evidence-based guidance, and credible support for pet owners and critically ill patients undergoing end-of-life care [48]. Some models have been developed in the area of human care and adapted for use in veterinary medicine, such as guided conversations about care goals and the communication of bad news [52]. Advance care directives serve to anticipate decisions that may arise during periods of acute illness crisis, which can be emotional and distressing for the owner [49].

In order to provide optimal symptom management of cancer patients, it is important to take a holistic approach considering any concurrent diseases and treatments in addition to the primary diagnosis and to anticipate and better manage disease complications and adverse events that may affect the patient's quality of life [21].

### 4.1. Evaluation Forms in Palliative Care

Symptom assessment process is a routine practice for palliative care providers, as they make it possible to recognize, diagnose, treat, and monitor the patient's symptoms [53, 54]. A multidimensional assessment of symptoms, focusing on severity, frequency, or distress generated by symptoms, provides more meaningful information about the impact of symptoms on the patient's quality of life [55].

Recognizing pain is essential for successful treatment, and some classic symptom assessment scales have been used in human palliative care, such as the McGill Quality of Life Questionnaire (MQOL), the African Palliative Care Outcome Scale (POS) (APCA), the Edmonton Symptom Assessment Scale (ESAS), and the Palliative Care Quality of Life Instrument (PQLI), among others [56]. In a comparative study of symptom assessment scales, including those mentioned above, it was found that more than 94% of the assessments classified the quality of life of patients in a multidimensional view, including physical, social, economic, spiritual, and emotional aspects and considered the patient's relationships with family and staff members [57, 58]. However, there is still no consensus on the ideal tools to assess quality of life in veterinary medicine. A limited number of studies using previously validated questionnaires, which were often associated with pain assessment, were found [59–61].

The Canine Symptom Assessment Scale (CSAS) was developed by the University of Pennsylvania for both clinical and research use. It was developed using dogs with solid tumors and their owners and was based on 12 predefined symptoms and two additional symptoms that the patient may have experienced in the past 10 days [62]. The evaluation of symptoms using this method is based on the notion that the client does not have theoretical or practical training to diagnose a clinical sign, a fact that does not invalidate the perception of a lay individual regarding the sensations or symptoms that the animal may present [62].

Currently, researchers assess quality of life in cats using a wide variety of tools, but only a few use instruments that have been validated [63]. One of them is the Cancer Treatment Form, which evaluates the quality of life of dogs and cats with cancer. It consists of 23 multiple-choice questions that assess aspects such as happiness, pain, appetite, hygiene, mental status, hydration, mobility, and overall health [64].

Other scales have already been used, such as the “HHHHHMM Quality of Life scale,” whose objective is to verify if the process after diagnosis was successful, as well as the necessary care during the follow-up of the animal in palliative care. In this scale, pain criteria, hunger, hydration, hygiene, happiness, and good and bad days are rated from 0 to 10 [65]. In order to evaluate pain secondary to cancer, a study was published in 2005 using a scale composed of 12 questions scored from 0 to 3 that covered the dog's emotional behavior, pain levels, appetite, fatigue, sleep problems, gastric problems, bowel problems, defecation, and urination [66]. Despite considering different criteria, both assessments are used to guide the veterinarian when recommending the euthanasia of a patient.

Some studies in the field of oncology evaluated the perspectives of the client regarding the quality of life of their animals during or after radiotherapy treatment [67, 68] and chemotherapy [69, 70]. Considering that caregivers of animals with serious diseases experience stress, anxiety, depression, and reduced quality of life, it is important that the veterinarian understands the potential for anguish and suffering in the owners as well as their animals [71]. This is also observed in studies carried out in the area of veterinary dermatology [72, 73].

In the year 2000, Laurel Lagoni and Carolyn Butler, who ran the Argus Center at CSU, adapted the subjective-objective-assessment (SOAP) to their medical records in order to evaluate the emotional state of caregivers [66].

### 4.2. Pet Owner Education in Veterinary Palliative Care

As the population of elderly animals grows, so does the interest of pet caregivers in providing quality veterinary care, enabling pets to age comfortably, with a better quality of life in the face of serious diseases. However, there is still a lack of knowledge regarding treatment options. In a survey involving more than 900 pet owners in the US, only 26% were aware of the availability of hospice and hospice care for animals at the end of life [10].

As the cultural view of companion animals continues to change, many people perceive pets as members of their families, with equal importance in relation to human individuals. This poses important ethical conflicts, as there may not be a clear distinction between the needs of the animal and the needs or desires of family members, which may lead to requests for futile interventions [3, 21].

Each caregiver reacts differently to treatment options. Some are interested in learning about home care, whether for convenience or economic reasons, while others are averse to medical procedures due to fear [39]. In fact, the illness of animals can result in intense emotional overload for owners, affecting their psychosocial functioning, and causing great stress, symptoms of anxiety and depression, and a reduced quality of life [71].

In a study of 99 owners of pets with cancer and 99 owners of healthy pets, it was found that depression and anxiety scores were significantly higher in owners of pets with cancer, who also exhibited greater anxiety in the face of the poor prognosis of their animals [74]. Thus, in addition to therapeutic support for patients, palliative care emerges as a tool for emotional support for clients and family members [16]. It should be noted that it is not the veterinarian's role to treat the client's mental health problems. However, their role in educating the client includes identifying signs of overload and suffering on the part of the caregiver. This allows veterinarians to understand the caregiver's perspective and seek appropriate tools to alleviate their suffering, such as psychological support or reviews of prescribed treatments [71].

For caregivers experiencing anticipatory, postmortem, or socially unrecognized grief, it is beneficial to name and normalize their grief, which can help them cope. Therefore, it is highly recommended that veterinary teams consider a multidisciplinary approach, utilizing the expertise of social workers and mental health professionals to provide support. This approach expands the unit of care, reassuring caregivers that it is normal to experience a range of emotions and that their feelings are supported and validated [75].

There are other factors that contribute to owners' feelings of distress. When an animal reduces its oral food intake, many owners interpret this as a rejection of love, as they equate food consumption with happiness and well-being. In addition, there is the fear that the animal will “die of hunger,” thus increasing the owner's feeling of suffering and guilt. Also, when the animal is not eating, the oral administration of drugs becomes more difficult. Therefore, caregivers begin to feel remorseful for not being able to provide medication to alleviate the pain. All these factors emphasize the concept of pets being part of the family [23]. Preparing pet owners by explaining the changes they should watch for and how to control the progression of the disease is essential. This proactive approach helps avoid unnecessary emergency room visits and hospitalizations [49].

Good communication with the owner is particularly important, as they are ultimately responsible for therapeutic decisions. Therefore, a negative perception on part of the owner may lead to noncompliance with treatment protocols or nonadherence to what has been recommended by the veterinarian, resulting in compromised patient care [76, 77].

When it comes to the death of a pet, the impact on its guardian can be comparable to the loss of a human being in psychological terms [78]. Thus, it is important that the veterinarian carefully considers how and when to recommend euthanasia and when to offer other therapies, such as hospices and palliative care [39].

Therefore, client education regarding end-of-life care is essential for VPC decision-making through timely, empathetic, and impartial communication, seeking to understand the caregiver's needs and goals for the pet [2]. The opportunity to discuss the diagnosis and prognosis with the owner in a compassionate manner builds a strong relationship of trust between all parties involved. This, in turn, facilitates decision-making for effective management by the veterinarian [49]. Such awareness depends on the extent to which care is embedded in an interdisciplinary team, so that the veterinarian, veterinary technician, and client can work together to develop an end-of-life care plan that reflects the goals and family preferences within the parameters of the best veterinary medical standards for the animal [34].

### 4.3. Interdisciplinary Veterinary Palliative Care Team

It has been observed that interdisciplinary team involvement is common in many palliative care settings around the world [9]. The team discusses and sets goals for the treatment of the patient jointly, everyone must be at the same hierarchical level, and there should always be a high degree of communication and cooperation between members [79].

In the context of a multidisciplinary team, several health professionals assess the patient individually, using resources from different fields without necessarily having to work as a team. However, it has been observed that a single professional cannot replicate the services carried out by a multidisciplinary team involved in providing palliative care [80]. This approach enables better control of symptoms, such as pain treatment, through the use of pharmaceutical compounds combined with modalities such as laser therapy, weight management, acupuncture, physiotherapy, therapeutic laser, thermal modification, massage, magnetic pulse therapy, and environmental modifications in the home, among others [34].  Table 1 provides a description of treatments presented in case reports of palliative care for animals with cancer.

Therefore, the organization of the team that will provide palliative care is crucial. One can list as important professionals in its composition: the veterinarian, technician or veterinary assistant, social worker, volunteers, chaplain or spiritual counselor, and grief counselor [1]. The formation of a human palliative care team sometimes includes the doctor, nurse, social worker, physical therapist, occupational therapist, complementary therapists, and chaplains [54]. Grief support can be offered through the approach of a family therapist or certified pet-loss and grief counselor [3].

One of the advantages of the interdisciplinary palliative care team is the set of different perspectives on the animal and its owner. Each professional in the team has a unique vision, collaborating in the integral care of the two as a unit of care. However, despite the existence of veterinary palliative care teams, psychological support is not often offered to owners of small animals or the staff involved in their care [1]. There is a lack of studies in the literature on veterinary palliative care services that include professionals in the field of psychology in their team who could offer support to the owners of these animals [4, 19, 21].

Evidence strongly suggests that human palliative care is best delivered through a multidisciplinary team approach [9]. Ideally, there would be a significant contribution from human health professionals in the follow-up of each clinical case attended. However, in some services, there is not enough coverage for social workers or psychologists to accompany all patients. At the Pain and Palliative Care Service of the Massachusetts Society for the Prevention of Cruelty to Animals, when available, a hospital social worker attends initial appointments or near the end of a visit [21]. Other professionals can also provide support to owners and veterinarians, such as chaplains, spiritual leaders, and/or grief counselors [1].

## 5. Palliative Care Protocols

The first consultation with owners of pets diagnosed with cancer can be challenging, both socially and medically [51]. Owners often experience stress due to a lack of understanding of their pet's illness and the concept of palliative care, as well as the emotional strain resulting from the patient's clinical decline and caregiver burden [39, 82]. This may lead to distressing questions about various aspects of their pet's cancer. Therefore, therapy should aim to minimize the financial, physical, emotional, and logistical burden on caregivers [56].

Attentive listening and asking open-ended questions assist in the initial approach, while the use of structured symptom questionnaires allows the veterinarian to gain a comprehensive understanding of the patient's clinical status without bias [62]. Listing symptoms and scoring them on a numerical scale is useful for identifying their severity and impact on the patient's quality of life. This approach also helps quantify changes in quality of life resulting from treatment response and disease progression [83]. Based on the severity and frequency of symptoms, pharmacological or nonpharmacological measures can be adopted for treatment. All prescribed medications should be reviewed to ensure that pet owners understand their use, frequency of administration, and possible side effects [38].

The palliative care consultation should be based on empathetic communication and shared decision-making, control of progressive symptoms, and advance care directives [49]. Some tools can be used to support end-of-life care planning, such as Go Wish decks [84], “Clarieta,” and “Patas na Mesa,” sold in Brazil. They are composed of approximately 20 to 30 cards that list end-of-life care preferences that the owner may express for their animal, such as availability of information, decision-making, priorities in symptom control, location of death, and care of the body.

Shearer developed the 5-step protocol for standardizing veterinary palliative care [38]. The protocol aims to cover all points necessary for the good practice of palliative care and suggests the following: (1) assessing the needs, beliefs, and objectives of the pet owner; (2) educating them about the illness process; (3) developing a personalized care plan for the pet and its owner; (4) applying palliative care techniques through written instructions and demonstration of the indicated care; and (5) promoting emotional support during the care and mourning process [38]. The fifth step involves continually checking in the palliative care relationship to see if caregivers are experiencing anticipatory grief, with the entire veterinary team playing a role in providing emotional and empathetic support to caregivers' suffering [75].

For each family structure, the demands of caring for an animal with cancer are different, as the intensity of care is related to the severity, time, and progression of the disease. Therefore, to determine the best course of treatment, it is necessary to understand the caregiver's needs, beliefs, and objectives through conversations regarding their financial, emotional, physical, and available time resources. Patient care will depend on the active involvement of the animal owner, who must be encouraged to ask questions and participate in care planning to verify what is viable and feasible for the treatment of their animal [75].

The development of a personalized care plan must enable the caregiver to care for their animal in a home environment. This involves assessing the caregiver's ability and willingness to assume specific care responsibilities, as well as evaluating the animal's acceptance and willingness to receive treatment. Structuring a detailed plan in writing and clearly explaining it to the animal owner is crucial. It is important to check their understanding of the plan, estimate the time and costs for daily and long-term care, and schedule consultations to monitor and reevaluate the animal's condition. Educating the caregiver in implementing the care plan should include instruction on various therapeutic techniques and evaluating the response to treatment, as well as any clinical worsening that their animal may experience [38, 75].

Animals with advanced disease often present with multiple symptoms that require joint assessment and management [85]. Each clinical sign of a cancer patient must be evaluated and treated according to its underlying cause, which may or may not be directly related to the disease. All treatments should only be chosen if the benefits produced are considered significant and superior to the risks or harms of the technique, optimizing care with a synergistic effect. A written daily treatment schedule or calendar allows pet owners and nurses to stay organized for needed care. Caregivers must note adverse events and responses to treatment on the calendar [39].

To define a hierarchy in the components of care, it can be considered that the basis of treatment is physical care, of acute and chronic symptoms. Afterward, assessing the patient's social well-being is necessary, focusing on their interaction with other animals and owners—finally, emotional well-being, which is defined by the patient's unique set of individual needs [2]. Therefore, the care plan must address the patient's physical, emotional, and social needs [75]. Recommendations from the veterinary literature and personal experience established the treatments described in Table 2. They can be adapted according to the animal's clinical condition, veterinary criteria, concomitant diseases, and owner availability. They may be associated with other necessary approaches and treatments and should not be followed as pre-established protocols.

The treatment of felines with cancer requires comprehensive care and management that takes into account the unique behaviors of the species. This involves providing safe and predictable environments that give them a sense of control and facilitating positive interactions with their caregivers. It is important to minimize stress, such as unpleasant-tasting medications or aggressive restraint, and reduce trips to the veterinarian by utilizing calming medications for travel. Encouraging the use of medications with preferred flavors, very small tablets, or transdermal treatments can be beneficial. Attention to treating feline symptoms of cancer should encompass environmental management and emotional enrichment, as these are essential ethical considerations, particularly regarding the principle of nonmaleficence in care [56].

## 6. Paradigms of Palliative Care Veterinary Practice

One of the major hurdles in delivering veterinary palliative care is the prevailing lack of awareness about this practice among both professionals and pet owners. The failure to refer patients early and provide these services presents significant challenges. The deeply ingrained culture of seeking curative treatments, developed over millennia, makes it challenging to accept that incurable conditions can be managed primarily for comfort, and that some therapies may come with undesirable consequences or may not be available for pets. However, guardians who are informed about this service are typically more engaged and motivated to adhere to the care plan [32].

The practice of euthanasia is widespread as a readily available option for treating incurable veterinary diseases, while it is not typically an option for human patients [88]. Euthanasia becomes a consideration when faced with deterioration in the animal's quality of life, or due to a lack of financial resources, time, and commitment from the owner, or even a lack of technical resources at the veterinary clinic. Moreover, euthanasia is also linked to the stress experienced by veterinary staff and owners [89], creating a significant imbalance in the care triad by failing to promote relief from suffering for all involved.

While mobile home euthanasia services are commonly available and accepted, services focused on palliative care remain inaccessible in many locations [32]. In palliative care, euthanasia can be viewed as one of the end-of-life care options guided by palliative principles. Therefore, comprehensive support for the patient's symptoms and family preparation is provided until the final decision is made, whether it involves euthanasia or not. The goal is to minimize suffering experienced by everyone involved when animals have compromised quality of life.

Palliated natural death can be facilitated through sedation, which occurs when the animal's quality of life is significantly impaired, and sedation is necessary to alleviate discomfort or pain. Following an interview, 5% of veterinarians expressed unconditional approval for this method, while 61% conditionally approved it, provided that pain could be effectively managed and that owners were willing and capable of providing adequate care [34].

While palliative care aims to manage patient symptoms such as pain, nausea, and anxiety through compassionate communication, caregiver education, and grief counseling services to owners [32], symptoms associated with natural death guided by palliative care can be distressing for many individuals. This includes veterinarians who may lack awareness of available pharmacological or nonpharmacological therapies for end-of-life care, or when resources are insufficient. Therefore, education and professional preparation are crucial to guide the development and dissemination of dignified care for veterinary patients with incurable diseases or those in the final stages of life.

## 7. Conclusion

Palliative care in veterinary medicine is a growing practice that benefits the professional staff, animals, and caregivers by providing a specialized service for the relief of pain and other clinical signs that lead to physical, social, and emotional suffering on the part of animals, and the social, emotional, and spiritual behavior of small animal owners. When possible, it should be performed by a multidisciplinary team and should extend from the diagnosis of cancer or other life-threatening illnesses up to and beyond the grief experienced by the client.

## Figures and Tables

**Table 1:** Therapeutic plan presented for the symptomatic treatment of animals with cancer in palliative care.

Animal	Diagnostics	Clinical findings	Proposed palliative care treatment	Owner's attitude towards palliative care	Article
Murphy Johnson, spayed female, Visla, 12 years old	(i) Splenic lymphoma past history: (i) Bilateral hip dysplasia (ii) Hypothyroidism (iii) Lower urinary tract infections (iv) Multiple dermal nodules (v) Eyelid melanocytomas (vi) Dental staining aging changes: (i) Energy decline (ii) Muscle atrophy (iii) Facial graying	(1) Initials: (i) Depression (ii) Weight loss (iii) Anorexia (iv) Severe lameness and inability to stand up —8 to 10% dehydration (v) Oral mucosa with hyperemia and petechiae (vi) Hemopistaxis (vii) Abdominal pain (2) In the first relapse, he presented: (i) Azotemia (ii) Kidney disease (iii) Paraneoplastic cachexia (3) Animal showed weight gain since the beginning of treatment. However, there was recurrence and metastasis to the liver, with (i) Sudden weight loss (ii) Azotemia (iii) Increased alkaline phosphatase (iv) Hypercalcemia (4) During the progression of the disease: (i) Poor body condition (ii) Cancer cachexia (iii) Muscle quivering (iv) Neurological signs (v) Mild seizure	(1) History of previous medications: (i) Deramaxx (ii) Dasuquin (iii) 3-V caps (iv) Soloxine (v) Meloxicam (vi) Gabapentin (vii) Tramadol (viii) Immunonutrition with Agaricus Bio, IP-6, canine platinum performance plus and ortho-chon (a pain control formula by platinum performance), and standard process (sp) musculoskeletal support (2) At the initial palliative care consultation: (i) Intravenous fluid therapy (ii) Ursodiol (iii) Amoxicillin with clavulanate (iv) Famotidine (v) Cerenia (vi) Palliative chemotherapy—personalized version of the modified Wisconsin protocol using vincristine and L-asparaginase and steroids, with doxorubicin dose fractionation (vii) Rx vitamin's onco support and hepato support (viii) Auburn lab's APF drops, which contain Siberian ginseng (3) Disease remission with relapse within 7 weeks (i) Dialogue with tutors about reducing the effectiveness of treatment (ii) Switching to COP protocol (vincristine, cyclophosphamide, and prednisone) with cytarabine and L-asparaginase (iii) Start of daily subcutaneous fluid therapy due to azotemia and kidney disease, with home administrations by the palliative care team when necessary, and supportive treatment with a local veterinarian (4) Second remission was achieved lasting 6 months. Conversation with guardians about third relapse (i) Mitoxantrone, lomustine, and dexamethasone protocol was started (5) Due to weight loss received: (i) Mirtazapine (ii) Periodic injections of nandrolone (iii) Depo-Provera intramuscularly (iv) Glutathione (v) ImmunoPro (purified whey) (vi) Hill's anorexia diet (vii) Lower her soloxine dose (viii) To support renal function: SP canine renal support azodyl, epitakin, and ultra EFA oil (OM-3 fatty acids) (ix) Bio sponge to control occasional bouts of loose stools (6) Fourth relapse with prior use of masitinib and metoclopramide (7) 17 months after diagnosis, the patient began end-of-life care (i) Diazepam for seizure control (ii) Nurse visits during hospice care (iii) Using miacalcin (calcitonin-salmon) (iv) End-of-life care planning: home euthanasia or palliative sedation with nalbuphine and diazepam to take her to hospital euthanasia (v) Opting for postmortem cremation	(i) Tutors refused immunophenotyping of splenic lymphoma, diagnosed by cytology, due to financial concerns (ii) The family wanted Murphy to be present at the wedding and chose to attempt a second remission of the disease (iii) Owners reported that Murphy's quality of life improved compared to previous years (iv) Family expressed satisfaction with Murphy's prolonged stable partial remission (v) Veterinary team exchanged text messages and phone calls during hospice	Villalobos [24]

Dolly, golden retriever, 14 years old	Nasal squamous cell carcinoma	(i) Chronic wound due to nonhealing incision at the biopsy site, with infected and necrotic tissue, putrid odor, and mucopurulent secretion (ii) Pain on touch-initial pain score of 2 to 3, on a simple descriptive scale (iii) After disease progression: hyporexia, reduction in activities, lethargy	Environment: office with feng shui design, pheromone diffusers, and music to minimize stress. Blanket for Dolly to lie down on (1) Assessed the owner's needs and beliefs (2) Education about the disease process: (i) Tutor teaching on how to assess quality of life (ii) Options to palliate cancer (iii) Manage disease side effects (iv) Prognosis (3) Therapeutic plan: (i) Maintenance of the use of firocoxib (ii) Addition of gabapentin and tramadol, and after worsening of pain, use of oxycodone (iii) Wei Qi booster e stasis breaker (Jing-tang do Dr. Xie) (iv) Yunnan Bai Yao for bleeding events (v) Laser therapy for pain control and healing (vi) Masitinib to slow cancer progression (vii) Palliative wound debridement to facilitate treatment and improve quality of life for Dolly and her owner (viii) Use of catheter for local anesthesia (ix) As the disease progresses, use continuous infusion of morphine, lidocaine, and ketamine before, during, and after wound hygiene (4) Written recommendations on how to administer medications and demonstration of wound cleaning (i) Carrying out euthanasia due to a decline in quality of life, with the presence of the owner and friends (5) Grief support: sending a condolence card and frequent contact	(i) Willing to do everything possible to preserve your dog's quality of life (ii) Agreed to the investigation of comorbidities in order to improve dolly's quality of life (iii) The dog was nervous away from her owner, which is why she did not agree to any hospitalization (iv) Open to alternative medicine options (v) Support network made up of a family member of the tutor, friends, and professionals (vi) Absence of financial barriers (vii) Euthanasia as a last resort if quality of life could not be maintained, I was hopeful that she would die at home in her sleep (viii) Would be cremated after death (ix) Tutor reported missing his dog, but believes that the last months of her life were more comfortable with the support of palliative care	Shearer [35]

Billy, neutered male, mixed breed, 14 years old	(i) Metastatic neoplasm: skin, liver, and splenic masses (ii) Development of stage 2 chronic kidney disease diagnosed within 6 months of initial palliative care consultation	(1) At the emergency consultation: (i) Acute lethargy (ii) Anorexia (iii) Mild anemia (iv) Mild lymphopenia (v) Moderate increase in liver enzymes (2) Later in the palliative care consultation: (i) Joint pain (3) Main symptoms in clinical evolution: (i) Hyporexia (ii) Pasty stools (iii) Lethargy (iv) Anxiety, night-time restlessness (v) Worsening pain	(1) Discussion of goals and implementation of an initial care plan with (i) Gabapentin; (ii) Supplements: fish oil, antioxidant; (iii) Acupuncture: dry needling (2) At the review appointment due to clinical worsening: (i) Gabapentin dose adjustment (ii) NSAID: carprofen, then meloxicam, then grapiprant (iii) Famotidine (iv) Maropitant (v) Metronidazole (vi) Pro-Pectalin (vii) Lorazepam (viii) HempRx (CBD oil) (ix) Supplement: continuity of the previous ones and probiotic (3) With the suspicion of an increase in abdominal masses, the following was added: (i) Amantadine (ii) Medication kit for comfort care, if needed at home: morphine and detomidine gel (4) Home euthanasia due to anorexia and neoplastic disease, 473 days after initial palliative care consultation	(i) Guardians did not wish to seek diagnoses or invasive procedures (ii) They chose to focus on comfort care (iii) Changes in the patient's condition altered care and diagnostic decisions, with a new blood test being performed six months after the initial consultation due to worsening hyporexia	Bennett and Cook [32]

Joe, Poodle, 16 years old	(1) Primary lung adenocarcinoma (2) past history: (i) Blind (ii) Overweight (iii) Inflammatory bowel disease (iv) Heart disease: grade IV/V heart murmur	(1) Initial signs: (i) Persistent cough (ii) Panting when walking (iii) Crackles in the caudal lung field (iv) Pneumonia (v) Nocturnal fever spikes (2) Progressively uncomfortable breathing, dyspneic (3) Authors report that Joe has enjoyed 22 weeks of good quality, stress-free, independent, and indulgent life since diagnosis (4) Hyporexia in his last week of life	(i) Rent of an oxygen concentrator and box, if oxygen support was needed at home (ii) Joe received full care at home during his illness journey (iii) As symptoms emerged, they reported that the timely and continuous support from the veterinarian and nurses managed to maximize Joe's comfort, even in the face of symptoms (iv) Ice packs for treating fever spikes (v) Variation of diet to make it palatable (vi) Euthanized at home	(i) Guardians chose palliative care over surgical care, not performing lung lobectomy (ii) The initial conversation for information about the diagnosis included several pauses in the veterinary speech, in response to the emotional reactions of the owners, in order to allow the assimilation of the content (iii) Treatment goal: maximize your comfort and independence at home for as long as possible (iv) The owners reported that they were pleased to be able to take care of Joe, spoil him, and enjoy his remaining life (v) For the euthanasia, the veterinarian went to the owners' home accompanied by all the nurses from the clinic who helped take care of Joe, creating a feeling of comfort for the owners, knowing that Joe was so loved (vi) They reported that the guardians' mourning was long, and 18 months later they found a way to accept that he had died, keeping his memory alive (vii) Owners were contacted by the veterinary team every 2 to 3 weeks to offer support and guidance (viii) After the animal passed away, they received a solidarity card to show the team's care, love, and sadness	Lam et al. [49]

Buddy, neutered male, west highland white terrier, 9 years old	Transitional cell carcinoma in his prostatic urethra	(1) The authors do not describe the initial symptoms of the disease (2) Report of chronic non-cancer-related low back pain 6 months after initial palliative care consultation (3) 2.5 years after diagnosis: (i) Rapid clinical decline (ii) Loss of interest in tutors, surroundings, and food	(1) Surgery to excise the tumor (2) Chemotherapy protocol with 5 sessions of doxorubicin (3) Use of metronomic piroxicam and doxycycline after completion of the injectable protocol of doxorubicin (4) For low back pain: (i) Acupuncture with dry needling (ii) Laser therapy (iii) Gabapentin (5) Tumor recurrence 1 year after diagnosis: (i) 4 carboplatin sessions (6) Euthanasia 2.5 years after diagnosis	(i) After treatment with carboplatin, the authors reported that the owners decided that they would not seek any aggressive palliative treatment option, focusing on quality of life (ii) Buddy's family opted for human euthanasia due to rapid clinical worsening, with a drop in quality of life score below 30/70	Downing, 2011 [81]

**Table 2:** Pharmacological and nonpharmacological therapies for the treatment of different clinical signs in pets with cancer.

Clinical sign	Pharmacological treatment	Nonpharmacological treatment
Pain	(i) Nonsteroidal and steroidal anti-inflammatory drugs: as indicated, choose the one for which the patient has greater tolerability (ii) Opioids: morphine, methadone, buprenorphine, tramadol, and fentanyl (iii) Anticonvulsants: gabapentin and Pregabalin (iv) Local anesthetics: bupivacaine, ropivacaine, and lidocaine (v) Paracetamol: exclusive use for dogs, should not be administered to cats (vi) N-methyl-D-aspartate (NMDA) receptor antagonists: amantadine (vii) Alpha 2 adrenergic receptor agonists (viii) Ketamine (ix) Cannabinoid: CBD (x) Antinerve growth factor monoclonal antibody (xi) Bisphosphonates	(i) Cold therapy (ii) Environmental modifications and assistance with ambulation (iii) Palliative radiation (iv) Omega 3 (v) Acupuncture (vi) Rehabilitation therapy (vii) Laser therapy (viii) Pulsed electromagnetic field therapy (ix) Stem cells (x) Such as platelet-rich plasma

Emesis	(i) Antiemetics: metoclopramide, ondansetron, maropitant, mirtazapine, and dolasetron (ii) Prokinetics: metoclopramide and cisapride Adjuvant therapies: (i) H2 receptor antagonists: famotidine and cimetidine (ii) Proton pump inhibitor: omeprazole	(i) Acupuncture

Seizures	(i) Anticonvulsants: potassium bromide, phenobarbital, gabapentin, felbamate, levetiracetam (ii) Intranasal diazepam for seizure control	

Mobility reduction		(i) Rehabilitation therapy (ii) Periodic change of position every 2 to 4 hours (iii) Use of padded surfaces, soft cushions, eggshell mattresses, waterbeds, with washable covers (iv) Preventive care of pressure ulcer formation (v) Pay attention to the animal's social welfare aspects (vi) Changes in the arrangement of food and water bowls, and litter boxes or toilet mats

Pressure ulcers	(i) Frequent cleaning of wounds and use of ointments and topical antibiotics (ii) Use of repellents (iii) Preventive skin care: use of hypoallergenic moisturizing creams based on urea or lactic acid	(i) Periodic change of position every 2 to 4 hours (ii) Use of padded surfaces, soft pillows, eggshell mattresses, and waterbeds, with washable covers (iii) Ozone therapy (iv) Laser therapy

Anorexia and cachexia syndrome	(i) Hormone: nandrolone decanoate (ii) Appetite stimulants: magestrol acetate, cyproheptadine, corticosteroids, metoclopramide, mirtazapine, and capromorelin (iii) Nutritional supplements: valine, leucine, and isoleucine; glutamine	(i) Offering highly palatable foods (ii) Omega 3 (iii) Offer of heated food (iv) Encouragement of moderate physical exercise (v) Rehabilitation therapy (vi) Feeding using probes force-feeding by placing food in a pet's mouth should not be recommended!

Cough	(i) Antitussive agents such as opioids: butorphanol and hydrocodone (ii) Steroids: fluticasone (iii) Mucolytic agents (iv) Bronchodilators: albuterol (v) Antibiotics if necessary for suspected infection	(i) Nebulization (ii) Rehabilitation therapy

Cold	(i) Use of laxatives: psyllium 2%, mineral oil, petrolatum, sodium dioctyl sulfosuccinate, magnesium salts, lactulone, and bisacodyl (ii) Glycerin suppository	(i) Wet feed supply (ii) Replacement of dehydration with subcutaneous fluids (iii) Encouragement of light physical exercise (iv) Pelvic massage (v) Enema

Dyspnea	(i) Opioids: morphine and codeine (ii) Benzodiazepines: midazolam (iii) Bronchodilators (iv) Nebulization with magnesium sulfate (v) Blood transfusion (vi) Palliative sedation	(i) Respiratory physiotherapy (ii) Oxygen (iii) Fans (iv) Thoracentesis for drainage of effusions

Sleep disorders	(i) Hormone: melatonin (ii) Antidepressants: amitriptyline and trazodone (iii) Benzodiazepines: clonazepam, diazepam, lorazepam, and oxazepam (iv) Antihistamine: diphenhydramine (v) Phenothiazines: chlorpromazine, and acepromazine (vi) Anticonvulsant: gabapentin and phenobarbital	(i) Encourage sleep hygiene habits: turn off the lights, keep the animal in a quiet environment, and find the place where the animal is used to sleeping (ii) Acupuncture (iii) Rehabilitation therapy

Ulcerated nodules	(i) Surgical removal (ii) Anesthetic block (iii) Antihemorrhagics: tranexamic acid (iv) Topical odor reduction treatment with activated charcoal, metronidazole, or aluminum hydroxide	(i) Daily dressing (ii) Cold saline solution and petrolatum compresses for bleeding wounds

Sources: [38, 39, 56, 75, 85–87]. The order of treatments described in the table does not correspond to a sequential indication for therapeutic practice.

## Data Availability

The authors declare that data sharing is not applicable to this article as no datasets were generated or analysed during the current study.
